# Collecting Data on the Social Determinants of Health to Advance Health Equity in Cancer Care in Canada: Patient and Community Perspectives

**DOI:** 10.3390/curroncol32070406

**Published:** 2025-07-16

**Authors:** Jacqueline L. Bender, Eryn Tong, Ekaterina An, Zhihui Amy Liu, Gilla K. Shapiro, Jonathan Avery, Alanna Chu, Christian Schulz-Quach, Sarah Hales, Alies Maybee, Ambreen Sayani, Andrew Pinto, Aisha Lofters

**Affiliations:** 1 Department of Supportive Care, Princess Margaret Cancer Centre, University Health Network, Toronto, ON M5G 2M9, Canada; eryn.tong@uhn.ca (E.T.); ekaterina.an@uhn.ca (E.A.); gilla.shapiro@uhn.ca (G.K.S.); christian.schulz-quach@uhn.ca (C.S.-Q.); sarah.hales@uhn.ca (S.H.); 2 Dalla Lana School of Public Health, University of Toronto, Toronto, ON M5T 3M7, Canada; ambreen.sayani@wchospital.ca (A.S.); andrew.pinto@utoronto.ca (A.P.); aisha.lofters@wchospital.ca (A.L.); 3 Department of Applied Psychology and Human Development, Ontario Institute for Studies in Education, University of Toronto, Toronto, ON M5S 1V6, Canada; 4 Department of Surgery, McMaster University, Hamilton, ON L8S 4L8, Canada; 5 Department of Biostatistics, Princess Margaret Cancer Centre, University Health Network, Toronto, ON M5G 2M9, Canada; zhihuiamy.liu@uhn.ca; 6 Department of Psychiatry, Temerty Faculty of Medicine, University of Toronto, Toronto, ON M5T 1R8, Canada; 7 Supportive Care, BC Cancer, Vancouver, BC V5Z 4E6, Canada; jonathan.avery@bccancer.bc.ca; 8 School of Leadership Studies, Royal Roads University, Victoria, BC V9B 5Y2, Canada; 9 School of Psychology, University of Ottawa, Ottawa, ON K1N 6N5, Canada; alanna.chu@uottawa.ca; 10 Patient Advisory Network, Toronto, ON M6P 2B7, Canada; alies.maybee@gmail.com; 11 Equity Mobilizing Partnerships in Community (EMPaCT), Women’s College Hospital, Toronto, ON M5G 1N8, Canada; 12 Women’s College Research and Innovation Institute, Women’s College Hospital, Toronto, ON M5G 1N8, Canada; 13 MAP Centre for Urban Health Solutions, Li Ka Shing Institute, St. Michael’s Hospital, Unity Health Toronto, Toronto, ON M5B 1W8, Canada; 14 Department of Family and Community Medicine, University of Toronto, Toronto, ON M5G 1V7, Canada

**Keywords:** cancer, social determinants of health, electronic health record, health equity

## Abstract

Despite advances in cancer care, disparities persist. The collection of the social determinants of health (SDOH) is fundamental to addressing disparities. However, SDOH are inconsistently collected in many regions of the world. This two-phase multiple methods study examined patient and community perspectives regarding SDOH data collection in Canada. In phase 1, a survey was administered to patients at a cancer centre (*n* = 549) to assess perspectives on an SDOH data collection tool. In phase 2, broader perspectives were sought through a community consultation with patient partners experiencing structural inequality (*n* = 15). Most participants were comfortable with SDOH data collection. Of survey respondents, 95% were comfortable with the collection of language, birthplace, sex, gender, education, and disability, and 82% to 94% were comfortable with SES, sexual orientation, social support, and race/ethnicity. Discomfort levels did not differ across subgroups, except women were more uncomfortable disclosing SES (OR: 2.00; 95%CI: 1.26, 3.19). Most (71%) preferred face-to-face data collection with a healthcare professional and only half were comfortable with storage of SDOH in electronic health records. Open-ended survey responses (*n* = 1533) and the community consultation revealed concerns about privacy, discrimination, relevance to care, and data accuracy. SDOH data collection efforts should include a clear rationale for patients, training for providers, strong data privacy and security measures, and actionable strategies to address needs.

## Simple Summary

Even though cancer care has improved, differences in care and outcomes still exist among different groups of people. One way to help reduce these differences is by collecting information about the social factors that affect health, known as the social determinants of health (SDOH). However, this information is not collected regularly in many parts of the world. This study, done in two parts, looked at what cancer patients and community members in Canada think about hospitals collecting SDOH information. Most people were comfortable with the hospital collecting SDOH data. However, patients and community members were concerned about privacy, discrimination, relevance to care, and data accuracy. To collect SDOH data in a way that works for patients, it is important to explain why the information is being collected, train healthcare staff, protect patient privacy, and have clear plans to use the information to improve care.

## 1. Introduction

While substantial progress in cancer screening, diagnosis, and treatment has led to reduced mortality rates for many cancers in Canada [1], disparities in morbidity and mortality persist [2,3,4,5]. These disparities are largely due to social, economic, and environmental disadvantages rooted in the social determinants of health (SDOH) [3]. The SDOH are the non-medical factors, such as income, social status, social support networks, employment, physical environment, gender, culture, racism etc., that determine individual and population health [6].

From screening to diagnosis to treatment, patients from socioeconomically disadvantaged communities in Canada experience worse cancer outcomes [7]. For example, lower-income and rural Canadians have a higher risk of developing and dying from cancer [3]. Recent immigrants and Indigenous people, many of whom live in low-income households, are less likely to report being screened for cancer [4]. Lower screening, higher incidence, and higher mortality rates have been reported in Black African-Caribbean and South Asian Canadians [5,8]. Additionally, two-spirit, lesbian, gay, bisexual, transgender and intersex people with cancer report more negative healthcare experiences and higher unmet needs [9].

The Canadian LaLonde Report set the stage in 1974 for understanding the key factors that determine health status [10]. Since then, evidence has shown that SDOH have a greater influence on health than genetic factors or access to healthcare services [6]. A fundamental strategy promoted by the World Health Organization to improve health equity is addressing the SDOH [11]. Screening for SDOH could help healthcare professionals tailor their care plans and connect patients to supports and services that improve outcomes and reduce health disparities [12]. Incorporation of SDOH data into electronic health records (EHR) could significantly enhance risk assessment and prediction to proactively identify and target patients who would most benefit from additional supports [13]. In addition, data on the SDOH could facilitate early detection and prevention of cancers, and inform policies, programmes, and practices to improve population health [12,14,15].

However, individual-level SDOH data are not collected in a systematic or standardized manner in many regions of the world, including in Canada [16,17,18], and the collection of SDOH data in cancer centres is not common [19]. While geographic area has been used as a measure of deprivation in Canada [20], U.S. studies have shown that individual rather than area-level data better predicts patient outcomes and needs [13]. The COVID-19 pandemic highlighted the structural inequities and gap in health equity data in Canada [21], leading several provincial health authorities to mandate the collection of SDOH data in all healthcare settings [22,23,24,25].

Broad interest-holder input is essential for the success of SDOH data collection efforts [26,27]. However, most research on SDOH data collection has focused on primary care, and patient views have been mixed. In Canada, less than 50% of 1,005 patients surveyed in 2011 [28], and less than 50% of 1306 patients surveyed in 2013 [29] considered SDOH collection important. Concern that the collection of SDOH could negatively affect their care was higher among females and minorities [28], which U.S. studies have found is associated with prior experiences with discrimination and racism in healthcare settings [30,31]. More recently, a qualitative study involving 27 primary care patients in 2019 found that patients would be comfortable sharing race and ethnicity information with their healthcare provider [32]. Likewise, a qualitative study of 13 internal medicine patients in 2022 [33] found general acceptance of SDOH collection, but uncertainty regarding its appropriateness in internal medicine given the limited time to address social needs.

A better understanding of cancer patients’ perspectives on the collection of SDOH is needed to guide implementation efforts in oncology settings in Canada. Using a standardized SDOH data collection tool [34] endorsed by a provincial health authority [35], we examined patient perspectives on SDOH collection in a cancer centre and captured broader perspectives through a community consultation with structurally marginalized patient partners. The findings offer practical insights for inclusive SDOH data collection practices in hospital settings.

## 2. Materials and Methods

### 2.1. Study Design and Setting

This was a two-phase sequential explanatory multiple methods study [36]. In phase 1, an online survey was administered to patients at the Princess Margaret Cancer Centre—an urban cancer care centre in Toronto Canada with over 18,000 new patient visits per year [37]—to assess their comfort with and perspectives on SDOH data collection using a standardized tool. In phase 2, guided by the Knowledge to Action framework [38] broader patient perspectives were sought through a community consultation with Equity-Mobilizing Partnerships in Community (EMPaCT) [39], a patient partner group with diverse lived and living experiences of social structural inequality. This study was reviewed and approved by the University Health Network Quality Improvement Institutional Review Board (QI ID: 21-0232). We followed the CHERRIES reporting guidelines for online surveys [40], which recommends reporting the view, participation, and completion rates. Given the multiple methods and data integration, we also relied on the Good Reporting of A Mixed Methods Study (GRAMMS) guidelines in the reporting of the results [41].

### 2.2. SDOH Data Collection Tool

To collect and assess perspectives on SDOH, we employed the Screening for Poverty And Related social determinations to improve Knowledge of and links to resources (SPARK) tool [34]. The SPARK tool was developed through a collaboration between several hospitals and a local public health organization in Toronto, Canada [42,43] and has since been adapted and adopted by a provincial health authority [23]. It consists of 8 demographic questions (i.e., language, born in Canada, indigenous identity, race, disabilities, sex at birth, gender identity, sexual orientation), 10 social needs questions (i.e., education, income/finances, food security, medication access, housing, transportation, phone and internet access, utilities, social supports, and employment), and 2 cultural questions (i.e., ethnicity and religion). The SPARK tool includes explanations for each question, how the data will be used, and definitions of terms. Adaptations to questions on sex, gender, and sexual orientation were made based on guidance from the Sexual and Gender Diversity in Cancer Care (SGDc) Program at the Princess Margaret Cancer Centre [44].

### 2.3. Phase 1—Patient Survey

#### 2.3.1. Participants

Individuals were eligible to participate if they were: (1) diagnosed with any type of cancer or an informal caregiver of a person diagnosed with cancer, (2) had an appointment, either in-person or virtual, at the Princess Margaret Cancer Centre in September 2021, (3) had an email address on file in their electronic health record (EHR), and (4) could read and respond in English. Caregivers were included because some patients include their caregiver’s email in the EHR instead of their own, and caregivers play an important role in helping patients navigate the healthcare system and complete forms.

#### 2.3.2. Data Collection

A study information letter and link to an anonymous online questionnaire hosted on the secure, web-based REDCap platform at the University Health Network was distributed by email to patients meeting eligible criteria between December 2021 and January 2022. The study information letter informed participants about the purpose of the study, that their participation would be anonymous and voluntary, what participation in the study involved, and how the data would be protected and used. Eligible participants were sent two follow-up email invitations.

The questionnaire was developed based on a study conducted by Lofters et al. [28] in 2011. Participants were first asked to indicate to what extent they agreed with the following statement: “I believe it is important for the hospital to collect personal information from patients to improve patient care.” The following definition of personal information was provided: “Information that includes your language preference, country of birth, race, long-term conditions, disabilities, sex, gender, sexual orientation, income/finances, employment, housing and social support.” Participants were asked to complete the SPARK tool [34]. For each question in the SPARK tool, participants were asked for their perspectives on: (1) their comfort with the hospital collecting this type of personal information (i.e., 1-very uncomfortable to 5-very comfortable); (2) whether they were able to answer the question with the response options provided (i.e., yes, no, not sure); and (3) feedback or suggestions for improvement of the question (open-ended). Participants were also asked to rate their comfort (i.e., 1-very uncomfortable to 5-very comfortable) with each of the following methods of data collection: (1) face-to-face with a healthcare provider, (2) face-to-face with a hospital administrative staff; (3) over the phone with hospital administrative staff; (4) over email with hospital administrative staff; (5) filling out a paper form at the hospital; (6) filling out an electronic form at the hospital; and (7) filling out a form online; and their comfort with their personal information being stored in their EHR. In addition, to assess experiences of discrimination, participants were asked to indicate whether they had been treated badly or unfairly in healthcare settings because of their personal characteristics (i.e., 1-never to 5-all the time). In one final open-ended question, participants were asked for their general feedback on SDOH collection by the hospital.

#### 2.3.3. Data Analysis

Descriptive statistics were used to summarize quantitative survey responses. Univariable and multivariable logistic regression was conducted to identify sociodemographic factors associated with discomfort with SDOH collection by the hospital, experiences with discrimination, and survey non-response. Binary outcome variables were created for this purpose: comfort (very comfortable, somewhat comfortable, and neutral vs. somewhat uncomfortable and uncomfortable); discrimination (never vs. rarely, sometimes, often, all of the time, do not know and prefer not to answer); and non-response (response vs. missing). Separate logistic regression models were generated to examine factors associated with discomfort with the SDOH variables where 5% or more reported discomfort (e.g., race, sexual orientation, education, finances, housing, employment, and social support). Covariates were identified a priori and included: born in Canada (yes vs. no), race (white vs. visible minority), gender (man vs. woman vs. other), sexual orientation (heterosexual vs. not-heterosexual), education (college/university vs. less), ability to make ends meet (yes vs. no), and housing (own home vs. rent, retirement home, long-term care, other). Odds ratio (OR) from univariable analysis and adjusted OR (aOR) from multivariable analysis with 95% confidence interval (CI) were reported. Variance inflation factor was calculated to assess multicollinearity. Statistical significance was defined as *p*-value < 0.05. Statistical analysis was performed in R, version 4.0.2 [45].

Open-ended survey questions for each SPARK question and a final general feedback question (*n* = 23 open-ended questions) were analyzed using reflexive thematic analysis [46] using NVivo version 10. Two coders (E.T. and E.A.) independently coded open-ended responses and generated initial codes. Through collaborative and iterative discussion between the coders, initial codes were compared and discussed to develop richer interpretations of the data. Codes were then collaboratively organized into a table of broader themes to develop “stories” and patterns of shared meaning across the dataset. E.T. and E.A. met with the senior author (J.L.B.) on a weekly basis to review ongoing analysis, discuss themes, and promote reflexivity.

### 2.4. Phase 2—Community Consultation

#### 2.4.1. Participants

EMPaCT members were invited to participate in the community consultation. Fifteen EMPaCT members diverse in age, gender, race, ethnicity, country of birth, sexual orientation, and experiences of disability and housing insecurity across various health conditions, including cancer participated. To avoid labelling members, more details about all EMPaCT members can be found at https://www.womensacademics.ca/empact/ (accessed 11 July 2025) where each person describes their identities and lived experience.

#### 2.4.2. Data Collection

Engagement and consultation of the EMPaCT community followed a structured, trauma-informed, co-designed process to increase safety, impact, and accountability in patient-oriented research [39]. This involved: (a) a 1-h scoping meeting with EMPaCT co-initiators (A.S. and A.M.) to clarify the purpose and desired outcomes of engaging with EMPaCT; (b) a 1-h preparatory meeting during which project team members received coaching from EMPaCT co-initiators on how to prepare and deliver the discussion slides to facilitate knowledge exchange; (c) a 1-h, audio-recorded community consultation with EMPaCT community members via videoconference, facilitated by EMPaCT co-initiators that centred on two questions: (1) What do you think of these results? (2) What would make people feel comfortable sharing their sociodemographic information with the hospital?

#### 2.4.3. Data Analysis

The EMPaCT community consultation was analyzed using reflexive thematic analysis [46] guided by a health equity framework focused on structural and social determinants of health [47]. Three coders (EMPaCT initiators A.S, A.M. and one community member) reviewed the transcript, jointly developed a code book, and independently coded the transcript. Codes were then compared and discussed and organized into broader themes. Preliminary findings were shared back with the community through a member-checking process to validate interpretations and ensure accuracy. This participatory approach ensures that the lived experiences of marginalized communities shapes both the findings and outcomes.

#### 2.4.4. Data Integration

Data from phases 1 and 2 were aligned, compared, and triangulated to generate a more comprehensive understand of patient and community views [48]. Data from phases 1 and 2 were integrated in the resulting recommendations.

## 3. Results

### 3.1. Phase 1—Survey Findings

#### 3.1.1. Survey Participant Characteristics

Of the 5090 survey initiations sent, 169 bounce backs and 5 death notifications were received. Of the 656 who initiated surveys, 549 returned completed surveys, representing a completion rate of 83.7%; view and participation rates were unavailable.

Of the 549 survey respondents, most were patients (93%), heterosexual (90%), white (77%), college or university educated (84%), did not have difficulty making ends meet (85%), and owned their house (74%). A roughly equal proportion of participants identified as men (50%) and women (49%) (Table 1).

#### 3.1.2. Comfort with SDOH Collection by the Hospital

Approximately three-quarters (73%) agreed that it was important for the hospital to collect personal information from patients to improve patient care. However, there were 45% (*n* = 248) did not respond to this question. Participants who were renting, living in a retirement home, or living in long-term care (aOR: 1.80; 95%CI:1.07, 3.04) were more likely to not respond to this question compared to those who owned their home.

**Figure 1:**
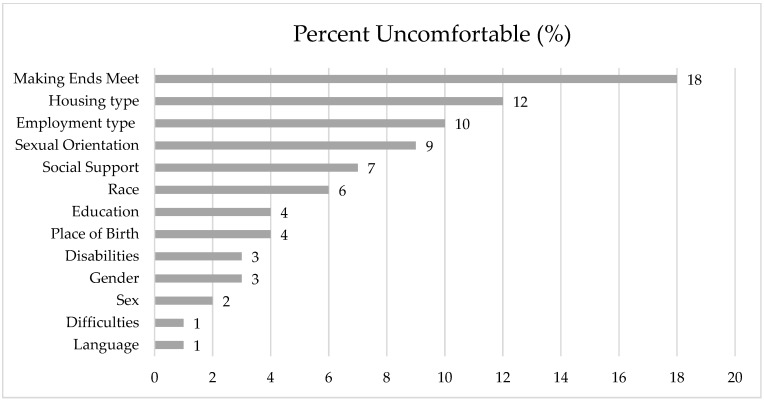
Percent uncomfortable with collection of SDOH variables by the hospital.

Over 95% of participants were comfortable with the collection of information about: language (lowest discomfort), place of birth, difficulties, disabilities, sex, gender, and education (Figure 1). In contrast, 5% or more were uncomfortable with the collection of information about: financial status (18%), housing (12%), employment (10%), sexual orientation (9%), social support (7%), and race (6%).

Discomfort with the hospital collecting information about race, sexual orientation, housing, employment, and social support was not associated with any of the sociodemographic variables examined (Table 2). However, women were more likely to be uncomfortable disclosing information about finances than men (aOR: 2.31; 95%CI: 1.23, 4.35).

#### 3.1.3. Comprehensiveness of Response Options

Over 90% of participants (92–98%) answered the questions with the response options provided, except for the question about physical/emotional difficulties, where 13% (*n* = 74) selected “not listed.” In open-ended feedback, participants explained that they experienced difficulties related to cancer and its treatment, which were not listed.

#### 3.1.4. Preferred Method of SDOH Data Collection

Most participants (77%, n = 415) were comfortable sharing their personal information face-to-face with an HCP, followed by filling out a form on the Internet (71%, n = 379) or on a tablet at the hospital (69%, n = 374). The fewest proportion of participants (62%, n = 328) were comfortable sharing personal information over the phone with hospital administrative staff.

#### 3.1.5. Comfort with Storage of SDOH in the EHR

Approximately half (57%, n = 200) were comfortable with the storage of their personal information in the EHR (57%, n = 200). However, 36% (n = 199) did not respond to this question. Men were more likely to not respond to this question than women (OR: 0.60; 95% CI: 0.42, 0.85). Participants who were renting, living in a retirement home, or living in long-term care (aOR: 1.91; 95%CI:1.13, 3.24) were more likely to not respond compared to those who owned their home.

#### 3.1.6. Experiences with Discrimination

Approximately one third (31%) reported feeling that they were treated badly or unfairly in the healthcare system due to personal characteristics. In the univariable analysis (Table 3), visible minorities (OR: 2.40; 95% CI: 1.56, 3.68), women (OR: 2.11; 95% CI: 1.44, 3.09), participants who had less than college/university education (OR: 0.52; 95% CI: 0.30, 0.91), and who had difficulty making ends meet (OR: 1.91; 95% CI: 1.14, 3.22) were more likely to report feeling they were treated badly/unfairly. In the multivariable model, being a visible minority (aOR: 2.63; 95%CI: 1.41, 4.91) and being a woman (aOR: 1.82; 95%CI: 1.15, 2.86) were associated with feeling badly/unfairly treated when other factors were considered.

#### 3.1.7. Participant Perspectives

Four overarching themes were identified through the thematic analysis of open-ended survey responses (*n* = 1533): (1) concerns about privacy and confidentiality; (2) concerns about bias and discrimination; (3) lack of clarity about relevance to care; and (4) concerns about accuracy and currency of information. These themes are described in further detail below and representative quotes are included in Table 4.

##### Theme 1: Concerns About Privacy and Confidentiality

Participants were more comfortable sharing their personal information if it was collected anonymously rather than linked to their identity and stored in their EHR. Participants raised concerns about the security of data storage in the EHR, namely because they were worried about the confidentiality of that information, and its accessibility by hospital staff and family members. Perceptions about the security of the data influenced their level of comfort with disclosing personal information. Similarly, participants voiced concerns about the circumstances around data collection, stating that “there is often very little privacy in clinic settings.” Concerns about privacy during data collection may be linked to patient preferences for verbal face-to-face data collection with a trusting HCP.

##### Theme 2: Concerns About Bias and Discrimination

Some of the concerns about privacy and confidentiality were related to concerns about bias and discrimination from hospital staff. Participants were concerned that disclosure of their personal information would result in unconscious bias and overt discrimination from hospital staff affecting the quality of their care. Participants were worried that hospital staff would look at them differently and treat them differently. Most who voiced these concerns did not identify as members of equity-seeking groups (e.g., they were White, well-educated, financially secure, etc.). Stemming from fears about bias and discrimination and its impact on their care, participants worried about whether the collection of SDOH would be mandatory. They recommended educating patients on the need for SDOH in the context of their care and how their personal information would be protected.

##### Theme 3: Lack of Understanding About Relevance to Care

Participants expressed frustration with the lack of clarity about the relevance of SDOH to their care. This theme was two-fold, in that participants expressed misunderstanding of equity and confused it with equality and secondly, they were concerned about how their personal information would be used. For the former, many participants emphasized the need to provide the same quality of care to all patients regardless of sociodemographic characteristics; some did not understand how sociodemographic characteristics could affect their care. For example, participants commonly expressed discomfort with disclosing financial information as they felt that this information was not needed by hospitals to provide care, and some were concerned that hospitals may use this information to withhold care or to require them to pay for their care. With regard to data use, respondents called for more transparency about how the data would be used by the hospital, with clear justifications for each sociodemographic data item.

##### Theme 4: Accuracy of Data

Participants also expressed concerns about the accuracy of the data. This stemmed from the recognition that personal information may change over time (e.g., housing type, financial status). Participants wanted assurances that providers would have access to accurate information about them and emphasized the need to keep their personal information up to date. Participants wanted a clear and timely mechanism for maintaining the accuracy of their personal information.

### 3.2. Phase 2—Community Consultation Findings

#### 3.2.1. Community Perspectives

Overall, EMPaCT community members confirmed that the survey findings resonated with their views and experiences. They felt that people would be more comfortable sharing personal information that is less stigmatized and less comfortable sharing personal information that could lead to stigma or discrimination. They raised the concern that providing personal information during hospital visits has the potential to bias healthcare providers, negatively impacting the healthcare patients receive, and further eroding trust in the healthcare system rooted in historical harms and traumas that continue to impact diverse communities. They suggested that diverse patients would be more likely to be comfortable sharing personal information when there is trust and continuity of care, coupled with potential immediate benefits such as social assistance or housing support. They also suggested that in a hospital setting, unlike a primary care setting, where patients are likely to encounter a range of healthcare providers with whom trust has not been established, there may be greater potential for harms associated with stereotyping. Four overarching recommendations were identified from the community consultation: (1) accountability, (2) transparency, (3) patient consent, (4) data accuracy.

##### Theme 1: Accountability

Community members were frustrated with the lack of action taken to improve the health of communities using data that was already available. They emphasized that healthcare providers and researchers need to understand and clearly communicate to patients how collecting personal information will create direct benefits for patients (e.g., access to social supports) or long-term benefits (e.g., care pathways for specific groups). As one community member explained, “If they can help me secure better housing, I would be happy to share this information with them.” Community members recommended the inclusion of patients in a data stewardship council to ensure the needs and benefits to patients are prioritized.

##### Theme 2: Transparency

Community members called for transparency and a clear policy that explains how personal information will be collected, stored, used, and for what duration. Additionally, community members emphasized that patients should be informed of how their personal information would be updated over time and by whom as some personal information may change over time. They recommended that a plan should be put in place for how this data will be kept up to date.

##### Theme 3: Patient Consent

It was unclear to community members how personal information would be stored and raised concerns about storage in the EHR providing healthcare providers whom they may not trust with unrestricted access to their information. They felt that patients should be able to provide tiered consent to access their personal information (e.g., consent for use of some data for specific purposes, and not others) and limit general access to specific clusters of data in EHRs. Additionally, they believed that patients should be informed of how they could remove their personal information from EHRs. They also recommended decoupling patient information collected for research purposes from ongoing clinical care to reduce the risk of stigma and discrimination.

##### Theme 4: Data Quality

Community members raised concerns about data accuracy, completeness, reliability, relevance and timeliness, which could lead to misinterpretations and further harm through policies enacted on false or inaccurate interpretations of missing data. They explained that some individuals may not feel comfortable providing the data requested and may decline to answer or provide false data. Patients who do not communicate well in English or who have low literacy may experience barriers to sharing the data requested. Community members recommended partnering with patients throughout the lifecycle of SDOH data collection and analysis to help understand some of the limitations, identify mitigating measures, and to assist with interpretation.

### 3.3. Recommendations

The findings from Phase 1 and Phase 2 were consolidated in the form of patient and community recommendations to guide the respectful, reliable, and inclusive collection of SDOH in healthcare settings (Table 5).

## 4. Discussion

### 4.1. Summary

There is increasing interest in the collection of SDOH to inform patient-centred care and improve health equity. This study examined patient perspectives on the collection of SDOH in a cancer centre and obtained the views of diverse patient partners from the community on the relevance and implications of the findings. Overall, comfort with the collection of SDOH by the cancer centre was high, ranging from 82% to 95%. Discomfort with SDOH data collection did not differ between participant subgroups, except women were more uncomfortable disclosing SES. However, participants raised concerns about privacy and confidentiality, bias and discrimination, relevance to care, and data accuracy. The community consultation validated the survey findings and offered important insights on strategies to ensure accountability, transparency, patient consent, and data quality.

### 4.2. Comfort with the Collection of SDOH That Is Less Stigmatizing

Comfort with the collection of SDOH was considerably higher in this sample of cancer patients than in a population-based survey of Canadian adults conducted 10 years earlier by Lofters et al. [28], suggesting growing public acceptance. However, comfort levels depended on the type of information collected. Like the study by Lofters et al., discomfort with SDOH data collection was lowest for language and highest for SES, though discomfort levels were much lower in the present study (e.g., 1% and 18% vs. 6.6% and 65%). Unlike the study by Lofters et al., discomfort levels in this study did not differ across subgroups, with the exception that women were similarly more uncomfortable disclosing SES. The lack of difference in comfort across subgroups may be related to the higher SES of the participants in the present study, as the samples were otherwise comparable in terms of the proportion of respondents who identified as female, born in Canada, and a visible minority. This finding suggests a possible positive intersectional effect of SES, whereby people of higher SES, regardless of other sociodemographic characteristics, may be more comfortable with SDOH in hospital settings. Regardless, these findings demonstrate that patients may be less comfortable sharing SDOH information if it could lead to stigma or discrimination.

### 4.3. Concerns About Discrimination and Impact on Care

Despite the high level of comfort with SDOH data collection, participants were concerned about privacy, discrimination, relevance to care, and data accuracy. Similar patient concerns have been reported in other Canadian studies [28,29,33]. The most common concern was the fear that SDOH data could lead to bias and discrimination by hospital staff negatively affecting their care. This was also the greatest concern reported by internal medicine patients in the study by Davis et al. [33]. Racialized patients in Davis’s study were fearful they would be racially targeted if they were asked these questions in the hospital. Nearly one-third of participants in the present study (31%) reported experiencing discrimination in the healthcare system, and those who identified as a racialized or as a woman were 2.6 and 1.8 times more likely to have experienced discrimination. A slightly lower proportion of participants in Lofters’ 2011 study [28] reported experiencing discrimination. However, concern that SDOH data could negatively affect their care was also higher among women and racialized minorities [28]. These findings mirror reports from the U.S. that show that women are more likely to experience discrimination in the healthcare system, most often related to race/ethnicity [49]. Problematically, SDOH data collection could place minoritized individuals at risk of excess stress which could exacerbate disparities [50,51]. Taken together, it is imperative that efforts to advance SDOH confront discrimination in the healthcare system; otherwise, there is risk of perpetuating inequities that these efforts are intending to address.

### 4.4. Preferred Method of SDOH Data Collection

A critical challenge to routinizing SDOH data collection in clinical practice, is determining how and who would bear the additional workload [19]. Survey participants’ and community members’ preferred method of SDOH data collection was with a trusted HCP. This finding is in line with previous studies of Canadian perspectives on SDOH data collection [28,29,33]. A trusting relationship with an HCP is perceived to facilitate disclosure and alleviate concerns related to stigma, discrimination, and privacy among in-patients in internal medicine [33]. Yet, there are concerns that SDOH data collection by HCPs may not be feasible or sustainable given the time required and the healthcare worker shortage [52]. However, implementation of routine SDOH screening in an oncology outpatient clinic in the U.S. which was conducted verbally by clinic staff (e.g., nurse navigator, social worker or medical student) and inputted directly into the EHR, was acceptable and did not increase visit time [19]. Self-administered technology-enabled data collection (e.g., through a web- or mobile app) may be another option and was the second most preferred data collection method among survey participants. Technology-enabled self-reported data could be conducted prior to the visit, integrated into the EHR, used at a point of care to support patients directly, and linked to health outcomes to measure and track heath equity indicators at the population level. While 71% of survey participants agreed with technology-enabled SDOH data collection, this survey was administered online to patients with email addresses on file and may represent a biased view of patients who have access to and are comfortable using communication technology and the Internet. Offering both high-tech (e.g., web-based self-report) and high-touch (e.g., collected verbally by a trusted provider) options for SDOH data collection would increase the reach and comprehensiveness of data collected, aligned with patient preferences.

### 4.5. Integration of SDOH in the EHR

Ultimately, for SDOH to be used to provide personalized care and improve population health, the data must be readily accessible and linked to patient health information. EHRs offer tremendous potential to aggregate, analyze, and integrate SDOH information across settings to improve patient care and population health [13,27]. A systematic review of the integration of SDOH information into EHRs by Chen et al. found that incorporating individual-level SDOH data led to improvements in referrals to a social worker, medication adherence, and a risk of hospitalization [13]. However, only 36% of survey participants in this study indicated they would be comfortable with the storage of their SDOH data in the EHR. Survey participants and community members alike cited concerns about the security of data storage in the EHR, the confidentiality of that information, and its accessibility by hospital staff and family members. Additionally, EMPaCT community members expressed concern about how truthful patients would be for fear of judgement and discrimination, resulting in inaccurate data. A scoping review of efforts to integrate SDOH information into EHRs in the U.S. and Canada by Wark et al. found that acceptability was lower among patient populations with low health literacy, in situations where the purpose was not communicated or understood, where screening questions were not actionable by the provider, and when screening administration and review processes did not allow for timely follow-up by the provider [53]. A subsequent systematic review of patient perspectives on integration of SDOH into EHRs in primary care by Caicedo et al., concluded that patients were more likely to accept the practice if they had trust in their healthcare providers, understood the reason, and there was an actionable outcome [54].

### 4.6. Desire for Action

An important finding that emerged from the community consultation was a desire for action. Community members indicated they would be more willing to share SDOH information if providers used it to address their needs. Similarly, in-patients in internal medicine were more likely to support SDOH data collection if hospital staff could provide meaningful community resource referrals, as they did not expect the hospital to intervene on their social needs [33]. Additionally, they expressed a need for hospital outreach and follow-up after discharge to help them access community resources. Similar findings were reported in the systematic review by Caicedo et al. which concluded that SDOH data collection should lead to actionable information along with follow-up appointments to ensure needs are addressed [54]. Some studies in the U.S. have examined how well providers address SDOH-related needs identified during SDOH screening. Lewis et al. found that providers could address 31% of needs but could find a diagnosis code for only 7% and billed for only 1% of them [55]. Another study reported that having a screener implement the SDOH screening helped providers identify the unmet needs of their patients which often led to new community partnerships to address those needs [56]. Implementation challenges included staff not understanding why SDOH were being screened, why they should screen for needs the provider could not address, and how to integrate the screener into workflows. Collectively these studies highlight an ethical imperative to ensure that SDOH efforts include strategies to use the data to improve patient care and address patient needs. Navigation services and community partnerships could be helpful in this regard. Patient navigators could collect, or screen previously collected SDOH data, identify resources in the hospital and community, and assist patients in accessing the necessary supports. Studies in the U.S. have begun to explore the role of the navigator in social needs screening and management with positive results [57].

### 4.7. Recommendations

The study findings demonstrate a need for patient and public education campaigns on the purpose and value of SDOH collection and clear communication, policies, and procedures that govern why SDOH data is being collected, how it is collected, who has access to it, how it will be used to benefit patients, and how it will be securely stored. This study revealed a lack of understanding regarding SDOH and how SDOH can affect health, illness and access to care, highlighting the importance of patient and public education campaigns. However, education and clear communication alone is not enough; equity-informed policies and procedures are needed to ensure SDOH data collection efforts are implemented safely, systematically, and equitably. Training HCPs and hospital staff in anti-oppression could help reduce the potential for harm. Anti-oppressive practice raises awareness of power, privilege and implicit bias and the psychological burden of equity-seeking groups from minority socialization and prior negative experiences [58]. SDOH data collection should be used to benefit patients directly by improving care and addressing needs, in addition to informing equity-oriented health policies, programmes, and practices to improve population health. To that end, HCPs must be equipped with the knowledge and skills and afforded the time to form trusting relationships with patients to alleviate concerns about bias and discrimination, facilitate disclosure of social needs, and understand and respond to them. Cancer centres implementing routine SDOH data collection efforts would benefit from leveraging navigation services and building community partnerships to help address patients’ social needs. Cancer centres must also put protocols, procedures, and infrastructure in place to ensure the privacy and security of the data. Lastly, patients and families should be engaged throughout the process to assist in data governance, implementation planning, and decision-making around policy initiatives to address health inequities.

### 4.8. Strengths and Limitations

While the number of individuals who initiated the survey (*n* = 656) represents a small proportion of patients who were sent an invitation (*n* = 5090) and the view rate is unknown, the survey completion rate was high at 83.7% (*n* = 549). The online survey and the self-selective nature of study participation may have introduced selection bias towards patients who are comfortable using the Internet and are interested in this topic. This may in part explain the higher SES of the sample. Hence, the findings may not represent the views of lower-income populations. Further, while 26% of survey participants identified as a visible minority, other studies conducted at the same cancer centre using in-person data collection methods suggest that the proportion of racialized patients receiving care at the cancer centre is 50% [59], which aligns with the proportion of area residents who identify as racialized [60]. Hence, the survey findings may not adequately represent the views of those who identify as Indigenous, racialized, or LGBTQS+. A more diverse sample may be less comfortable with SDOH data collection, in general, and responding to questions where they identify as a minority specifically [28,31]. Given the concerns participants raised about sharing potentially stigmatizing information, the anonymity of the survey could have increased participant comfort with disclosing SDOH in this study. We tried to address the limited racial, sexuality and gender, and economic diversity of the sample by collecting broader perspectives from a patient partner community with diverse lived and living experiences with structural marginalization. The collection and integration of quantitative and qualitative data from the patients in the cancer centre and feedback from EMPaCT members in the community engagement provided a rich understanding of the topic from multiple perspectives. Lastly, the higher non-response associated with questions about SDOH, in general, and storage in the EHR in comparison to questions about specific types of data, is a limitation, but provides further evidence that patient comfort with SDOH varies based on the type of data collected.

## 5. Conclusions

Most participants in this sample of Canadian patients with cancer were comfortable with routine SDOH data collection by the cancer centre. However, concerns were raised about relevance to care, privacy, discrimination, and data security that were validated by community members with experience with social structural inequality. Efforts to implement SDOH in cancer centres should include a clear rationale, provider training, data privacy and security measures, and actionable strategies to address patient needs while confronting discrimination in the healthcare system.

## Figures and Tables

**Table 1:** Participant characteristics (*n* = 549).

Characteristic	*n* (%)
Participant (*n* = 454)	
Patient	507 (93)
Caregiver	36 (7)
Born in Canada (*n* = 519)	324 (62)
Sex assigned at birth (*n* = 541)	
Male	272 (50)
Female	269 (49)
Gender (*n* = 544)	
Man	270 (50)
Women	267 (49)
Non-binary/Two-spirit	2 (0)
Not listed	5 (1)
Sexual orientation	
Straight (heterosexual)	451 (90)
Lesbian/gay/bisexual/queer/questioning	32 (6)
Asexual	20 (4)
Not listed	1 (0)
Indigenous * (*n* = 528)	7 (1)
Race **	
White	425 (77)
East Asian	40 (7)
Black	27 (4)
South Asian/Indo-Caribbean	18 (3)
Southeast Asian	14 (3)
Latin American	10 (2)
Middle Eastern	3 (1)
Indigenous	3 (1)
Not listed	15 (3)
Language translation preferred (*n* = 468)	25 (5)
Highest level of education (*n* = 536)	
Less than high school	15 (3)
Completed high school	43 (8)
Trades certificate/diploma	29 (5)
College/University	449 (84)
Difficulty making ends meet (*n* = 475)	73 (15)
Physical, mental or emotional functional limitations *	373
Disabilities *	396
Housing (*n* = 518)	
Own home	385 (74)
Renting home	113 (22)
Shelter/couch surfing	7 (1)
Retirement home/long-term care	3 (1)
Not listed	10 (2)
Casual, short-term, temporary employment (*n* = 521)	42 (8)
Available social support (*n* = 516)	474 (92)

* Cells with counts with less than 10% of the sample were excluded from data analysis and reporting. ** Participants selected multiple responses if relevant.

**Table 2:** Factors associated with discomfort with SDOH collection by the hospital.

Covariate Comparisons	Race		Sexual Orientation		Finance		Housing		Employment		Social Support	
	Univariable	Multivariable	Univariable	Multivariable	Univariable	Multivariable	Univariable	Multivariable	Univariable	Multivariable	Univariable	Multivariable
	OR (95% CI)	aOR (95% CI)	OR (95% CI)	aOR (95% CI)	OR (95% CI)	aOR (95% CI)	OR (95% CI)	aOR (95% CI)	OR (95% CI)	aOR (95% CI)	OR (95% CI)	aOR (95% CI)
Not born in Canada vs. born in Canada	2.11 (0.77–5.81)	3.68 (0.81–16.65)	0.65 (0.34–1.26)	0.99 (0.33–2.98)	1.20 (0.74–1.94)	1.11 (0.53–2.31)	1.00 (0.56–1.78)	1.10 (0.42–2.84)	0.93 (0.50–1.72)	0.85 (0.32–2.29)	0.81 (0.39–1.70)	0.58 (0.17–1.99)
White vs. Visible Minority	1.11 (0.40–3.13)	2.87 (0.69–11.99)	0.67 (0.29–1.54)	0.27 (0.03–2.33)	1.18 (0.70–2.00)	1.25 (0.53–2.95)	1.37 (0.75–2.50)	1.66 (0.58–4.72)	0.93 (0.45–1.94)	1.17 (0.37–3.76)	1.15 (0.50–2.64)	1.29 (0.32–5.26)
Man vs. Woman vs. Non-Binary	0.79 (0.35–1.78) 3.02 (0.34–26.86)	0.45 (0.15–1.41)	1.45 (0.79–2.68)	1.18 (0.46–3.01)	2.00 (1.26–3.19) 4.69 (0.75–29.10)	2.31 (1.23–4.35)	1.58 (0.92–2.71) 3.21 (0.32–32.06)	1.42 (0.65–3.14)	1.12 (0.62–2.02)	0.93 (0.40–2.13)	1.46 (0.72–2.96) 4.48 (0.47–42.80)	0.76 (0.26–2.26)
Heterosexual vs. Lesbian/Gay/Bisexual/Queer/Questioning	0.72 (0.17–3.17)	0.50 (0.06–4.20)	1.41 (0.47–4.22)	2.13 (0.58–7.86)	0.66 (0.29–1.52)	0.54 (0.16–1.84)	1.79 (0.82–3.89)	2.62 (0.96–7.13)	1.82 (0.80–4.14)	2.35 (0.79–7.01)	0.84 (0.25–2.86)	1.18 (0.24–5.72)
≤College/University vs. <College/University	0.40 (0.09–1.74)	0.36 (0.05–2.93)	0.50 (0.18–1.45)		1.32 (0.74–2.34)	0.99 (0.41–2.36)	1.14 (0.57–2.29)	0.93 (0.30–2.84)	1.15 (0.54–2.47)	0.67 (0.19–2.38)	1.82 (0.82–4.02)	0.84 (0.18–3.94)
Making Ends Meet vs. Difficulty Making Ends Meet	1.15 (0.38–3.49)		0.50 (0.15–1.69)	0.52 (0.06–4.35)	1.08 (0.52–2.24)	0.94 (0.37–2.43)	1.57 (0.72–3.44)	1.01 (0.30–3.46)	1.30 (0.52–3.29)	1.58 (0.44–5.66)	1.98 (0.76–5.19)	1.05 (0.18–5.97)
Own home vs. Rent/Retirement Home/Long-term care vs. Couch surfing/Shelter	1.57 (0.63–3.96) 1.43 (0.18–11.45)	1.45 (0.41–5.20) 4.21 (0.45–39.74)	1.04 (0.48–2.28) 0.70 (0.09–5.42)	0.86 (0.23–3.22)	1.36 (0.80–2.33) 0.31 (0.04–2.38)	1.08 (0.50–2.35) 0.58 (0.07–4.84)	1.17 (0.58–2.33) 1.23 (0.27–5.56)	0.57 (0.17–1.85) 1.99 (0.37–10.81)	0.76 (0.33–1.79) 1.42 (0.31–6.46)	0.26 (0.05–1.26) 1.76 (0.31–10.07)	1.20 (0.49–2.90) 1.06 (0.13–8.41)	0.62 (0.12–3.25) 1.76 (0.18–17.22)

**Table 3:** Factors associated with experiencing discrimination in healthcare settings.

Covariate Comparisons	Univariable		Multivariable	
	OR (95% CI)	*p*-Value	aOR (95% CI)	*p*-Value
Not born in Canada vs. born in Canada	0.71 (0.48–1.05)	0.086	1.22 (0.70–2.11)	0.49
White vs. Visible Minority	2.40 (1.56–3.68)	<0.001	2.63 (1.41–4.91)	0.0023
Man vs. Woman vs. Non-Binary	2.11 (1.44–3.09) 3.37 (0.66–17.12)	<0.00 0.14	1.82 (1.15–2.86)	0.0099
Heterosexual vs. Lesbian/Gay/Bisexual/Queer/Questioning	1.75 (1.00–3.06)	0.051	1.94 (0.99–3.83)	0.055
≤College/university vs. <College/University	0.52 (0.30–0.91)	0.022	0.66 (0.34–1.28)	0.22
Making Ends Meet vs. Difficulty Making Ends Meet	1.91 (1.14–3.22)	0.014	1.14 (0.58–2.28)	0.7
Own Home vs. Rent/Retirement home/Long-term care vs. Couch surfing/Shelter	1.42 (0.91–2.22) 1.51 (0.54–4.26)	0.13 0.43	0.92 (0.51–1.66) 1.23 (0.32–4.71)	0.78 0.76

**Table 4:** Representative quotes of survey participant perspectives.

Themes	Representative Quotes
Theme 1: Concerns about privacy and confidentiality	
Subtheme: Concerns about data security	“My only concerns would be: 1. Providing security against hackers. 2. Providing security against anyone accessing this information who is not authorized to do so. 3. Providing these information records to the patient if requested and having a process to correct any information that is wrong or misleading.” (P134)
Subtheme: Concerns about linking data to personal health record	“I support the anonymous collection of personal information however I would not want any of my answers to be tied to my identity.” (P113)
Subtheme: Concerns about data collection methods	“There is no privacy when hospital staff repeats personal information orally. There should be a screen they can show for patient to review and verify without everyone in the room hearing where you live and other personal info.” (P472)
Theme 2: Concerns about bias and discrimination	
Subtheme: Bias	“It may be perceived negatively by some staff who would be unconsciously biased regarding the client.” (P241)
Subtheme: Concern about mandated data collection	“There would need to be understanding for those who are not comfortable. Measures taken to help everyone believe it doesn’t harm them or their care.” (P629)
Theme 3: Lack of clarity about relevance to care	
Subtheme: Equity vs. equality	“The hospital needs only to know these topics for medical reasons. Race, age, and sex and emotional support structure affect health. Identifying these characteristics to ensure some equity for race, gender, or whatever is ridiculous. Equality of treatment is good. Equity in outcome is not attainable.” (P118) “This has nothing to do with the obligation of hospitals to provide the same healthcare irrespective of who you are, what you look like and whether you cuddle up to your cat, dog or partner at night. Who cares?” (P421)
Subtheme: Concerns about data use	“I don’t really see the need for an assessment of that level of detail. The submission of personal information should only be requested if clear purpose is provided. These broad questions don’t really help to understand why this information is needed and how it’ll be used. Without that clarity I would be uncomfortable.” (P629)
Theme 4: Accuracy and currency of information	
	“Concern about possible data collection errors and outdated or changing information. I’d like to know if the data stored is ever changeable in terms of my beliefs which may change over time, my trust in the institution’s security, etc.” (P211) “Since this information will change continuously having it as part of a permanent record has little value with more possibility of damage.” (P15)

**Table 5:** Recommendations for inclusive SDOH data collection.

	Recommendations
1	Establish a data stewardship council of diverse patient partners to provide oversight
2	Co-design data collection and use policies and procedures that prioritize patient needs
3	Clearly communicate who will access the data, for what purposes, for what duration, and the potential benefits and risks of data collection
4	Clearly explain each question, why it is being asked, and how the data will be used
5	Prioritize plain language to improve health literacy and resource accessibility
6	Minimize the potential for harm by ensuring that all staff involved in data collection and interpretation have been trained in culturally sensitive, anti-oppressive, trauma-informed practice
7	Provide supports and culturally appropriate services to help manage any potential harm from data collection
8	Establish protocols, processes, and infrastructure to protect patient privacy during data collection (e.g., in a private space, using secure online methods) and confidentiality during data storage and use (e.g., data security, authorized access)
9	Establish procedures to allow patients the opportunity to provide ongoing consent and the autonomy to update, revise or remove data
10	Mitigate bias in reporting based on false or missing data by identifying and addressing inaccuracies to ensure accurate and valid data.
11	Ensure data collection includes strategies to improve patient care and address patient social needs.

## Data Availability

Data are available from the corresponding author for eligible researchers according to institutional procedures upon request.

## Abbreviations

The following abbreviations are used in this manuscript:

SDOH	Social Determinants of Health
EHR	Electronic Health Record
HCP	Healthcare provider
SES	Socioeconomic status
EMPaCT	Equity-Mobilizing Partnerships in Community
SPARK	Screening for Poverty And Related social determinations to improve Knowledge
